# A pilot study on psychosocial factors and perceptions of organizational health among a sample of U.S. waste workers

**DOI:** 10.1038/s41598-024-59912-9

**Published:** 2024-04-22

**Authors:** Aurora B. Le, Abas Shkembi, Shawn G. Gibbs, Richard L. Neitzel

**Affiliations:** 1 Department of Health Behavior, School of Public Health, Texas A&M University, 212 Adriance Lab Road (TAMU 1266), College Station, TX 77843 USA; 2 Department of Environmental Health Sciences, School of Public Health, University of Michigan, Ann Arbor, MI USA; 3 Department of Environmental and Occupational Health, School of Public Health, Texas A&M University, College Station, TX USA

**Keywords:** Organizational health, Psychosocial factors, Solid waste, Waste workers, Psychology and behaviour, Public health

## Abstract

Solid waste workers encounter a number of occupational hazards that are likely to induce stress. Thus, there are likely to be psychosocial factors that also contribute to their overall perceptions of organizational health. However, attitudes regarding the aforementioned among solid waste workers’ have not been assessed. This descriptive, cross-sectional pilot study operationalized the INPUTS Survey to determine workers’ perceptions of organizational health and other psychosocial factors of work. Percentage and mean responses to each INPUTS domain are presented in accordance with their survey manual. Pearson’s chi-squared tests were run on count data; Fisher’s exact tests were run for count data with fewer than five samples. ANOVAs were run on the continuous items. Due to a relatively low sample size (N = 68), two-sided *p* values < 0.1 were considered statistically significant. Most solid waste worker participants reported high decision authority, that they perceived their management to prioritize workplace health and safety, and had high job satisfaction. However, perceptions of support for health outside of the realm of occupational safety and health was lower. Addressing traditional occupational health hazards continues to take precedence in this industry, with less of a focus on how the social determinants of health may impact workplace health.

## Introduction

Despite their being nearly half a million workers in waste management and remediation in the United States (U.S.), they remain an understudied worker population in in occupational safety and health (OSH) research. This worker population has higher-than-average total recordable injury and illness rates compared to workers across all private industries (3.1 vs. 2.7 per 100-full time workers). Most research on waste workers—of any type—has heavily focused on informal waste workers in Asia, Africa, and South America^1–7^. In the U.S., solid waste workers, who manage non-hazardous residential and commercial waste, comprise the majority of this industry^8^. Due to the nature of their work, solid waste workers are exposed to the spectrum of traditional occupational hazards—such as biohazards (e.g., human and animal fluids and excreta, bioaerosols), chemical exposures such as volatile organic compounds and improperly disposed chemicals, physical hazards like noise, poor ergonomics, injury risks, and environmental conditions (e.g., extreme weather, smog, pollution)—which not only affect their physical health outcomes but also mental health outcomes^9–11^.

In OSH research and practice, there is growing recognition that workers’ health, safety, and well-being are affected by more than the aforementioned traditional occupational health hazards. The bidirectional influence of physical and mental health is well known but received greater attention in the workplace, especially for essential and frontline workers, during the COVID-19 pandemic^1,14–16^. In recent decades, the contribution of psychosocial factors of work on employee health has been increasingly researched and validated^12,13^. Psychosocial factors of work are the social, organizational, and managerial features of a job that can result in physical and/or mental health impacts when taking into account a worker’s feelings, attitudes, behaviors, and physiology^12,13^. Adverse working conditions—such as high demands, low job autonomy, perceptions of minimal supervisor and/or coworker support, and job dissatisfaction—are all examples of psychosocial factors that can cause adverse health consequences. These consequences can include heightened stress, poorer occupational safety outcomes (e.g., higher injury rates, more frequent accidents), and other negative health outcomes (e.g., greater cardiovascular disease risk, higher susceptibility to musculoskeletal disorders, sleep disorders, gastrointestinal issues)^12,13,17^.

Another factor that has been well-documented to affect workers’ health outcomes (e.g., injury, illness) is organizational health. Organizational health describes aspects of the working environment related to the employees’ health; this can include occupational stress, the well-being of the employee in the working environment, leadership styles, and the relationship between management, operations, strategy, and culture. The state of the organization may be beneficial (i.e., promoting health) or burdening (i.e., causing illness) for workers^18–21^. Consequently, organizational health can impact psychosocial factors of work, such as resulting in job satisfaction or dissatisfaction, high or low turnover intention, and positive or negative perceptions of supervisor support, which in turn can affect an individual's health outcomes.

Given the known occupational risks that solid waste workers encounter, there are likely to be psychosocial factors that also contribute to their overall perceptions of health. Further, perceptions of organizational health may vary substantially at each workplace due to organizational (e.g., site-specific management practices, differing levels of supervisor and co-worker support, and/or job demands and resources)  which can influence psychosocial factors at work^12,18,19^. However, to the best of our knowledge, neither attitudes regarding U.S. solid waste workers’ psychosocial environment nor their attitudes regarding organizational health have been explored. We aim to characterize perceptions of organizational health and other psychosocial factors of work among a sample of U.S. solid waste workers in this descriptive pilot study.

## Methods

### Study participants

Adult solid waste workers aged 18 and above were recruited by contacting the site supervisors of 39 solid waste sites using of publicly available contact information. Workers from three solid waste sites in Southeast Michigan participated in this cross-sectional study in the fall of 2021. The three sites in the study are characterized as: (1) a single-site, family-owned, small-scale waste disposal site that provided hauling services and had all onsite workers participate in the study; (2) a single-site county-level waste management authority that only provided recycling services and had all onsite worker participate in the study; and (3) a multi-site, large-scale industrial waste management authority that contained both hauling and landfill divisions where approximately half the onsite workers participated in the study. Only the workers in the large-scale industrial waste management company were unionized, which left the majority of the study participants not unionized. After providing informed consent, participating volunteers were asked to provide information on occupational biohazard exposures, work stress levels and their effects, psychosocial factors of work, and organizational health. For compensation, study participants received $40.

### Questionnaire

The survey instrument utilized for this study, the Center for the Promotion of Health in the New England Workplace (CPH-NEW) Health and Safety Climate INPUTS Survey, has not been previously operationalized with this worker population. INPUTS, developed in 2011, has been used to measure workplace characteristics associated with employee health outcomes across all industries; items use existing validated instruments (e.g., Job Content Questionnaire, Work-Family Conflict), condensed for the purpose of providing an overview of the organization’s health. Specifically, the INPUTS survey was “designed to provide an overall assessment of workforce attitudes related to the physical and psychosocial work environment, including factors that support or detract from a healthy worksite culture”^22^. Hence, we sought to explore solid waste workers’ perceptions of their psychosocial factors of work and organizational health by operationalizing this survey for this pilot study.

Each participant was administered a survey at the beginning or end of their shift, which took approximately 20 min to complete. The 74 questions in the instrument included demographics, perceived biohazard exposure and training resources^23^, effort-reward imbalance^24^, and assessed worker attitudes and factors related to the psychosocial work environment that contribute to organizational health. This article focuses on the findings from the last portion of the survey. The INPUTS Survey^22^ was operationalized to determine a workplace’s health and safety climate. The survey is meant to be aggregated at the organizational level, not to examine differences at an individual worker-level. The survey contains 23 items spanning 19 domains such as management health and safety support, coworker support, work-family conflict, etc. To streamline the findings, the 19 domains were grouped into four main categories: (1) psychosocial environment and ergonomic risk; (2) interpersonal relationships; (3) organizational health and safety factors; and (4) overall job perceptions.

### Questionnaire items

As outlined by CPH-NEW, for all items, the higher the value, the healthier the work environment. Unless otherwise noted, all items were asked on a point Likert scale of “strongly disagree” to “strongly agree”. Job demands, skill discretion, and decision authority for each job site were measured by assessing workers’ decision authority, psychological demands, skill discretion, and ergonomic risk. Interpersonal relationships by job site were measured by assessing coworker and supervisor support, work-family and family-work conflict, and coworker health climate. Organizational health and safety factors by job site were measured by management health and safety support, company health support, supervisor health climate, organizational health climate, and employer-provided health opportunities. Job perceptions by job site were measured by overall satisfaction, whether employees would recommend working at their organization, commute time, and workplace safety. Table 1 presents each item-response category in detail.

**Table 1:** Item-response categories of CPH-NEW INPUTS Survey.

Item	Detail	Possible responses	Outcome
Job demands, skill discretion, and decision authority			
High decision authority	“My job allows me to make a lot of decisions on my own”	4-point Likert scale	% “agree” and “strongly agree”
Low psychological demands	“My job requires me working very hard”	4-point Likert scale	% “strongly disagree” and “disagree”
High skill discretion	“My job requires me to be creative” and “My job requires a high level of skill”	4-point Likert scale	Total % “agree” and “strongly agree” from both items
Low hand motion ergonomic risk	“My job regularly requires me to perform repetitive or forceful hand movements”	4-point Likert scale	% “strongly disagree” and “agree”
Interpersonal relationships			
Coworker support	“The people I work with take a personal interest in me” and “The people I work with can be relied on when I need help”	4-point Likert scale	Total % “agree” and “strongly agree” from both items
Supervisor support	“My supervisor is concerned about the welfare of those under him or her” and “My supervisor is helpful in getting the job done”	4-point Likert scale	Total % “agree” and “strongly agree” from both items
Low work-family conflict	“How often do things going on in the workplace make you feel tense and irritable at home?”	5-point Likert scale (never, occasionally, sometimes, often, and most of the time)	% “never” and “occasionally”
Low family-work conflict	“How often do things going on at home make you feel tense and irritable on the job?”	5-point frequency scale (never, occasionally, sometimes, often, and most of the time)	% “never” and “occasionally”
Coworker health climate	“If my health gets worse, my coworkers would support my recovery” and “My coworkers would support my use of sick days for illness or mental health”	5-point Likert scale, including “neutral”	Total % “agree” and “strongly agree” from both items
Organizational health and safety			
Management health and safety support	“In the facility, management considered workplace health and safety to be important”	4-point Likert scale	% “agree” and “strongly agree”
Company health support	“Overall, how supportive is your company of your personal health?”	Scale of 1 (extremely unsupportive) to 10 (extremely supportive)	Mean value
Supervisor health climate	“My supervisor encourages healthy behaviors”	5-point Likert scale, including “neutral”	% “agree” and “strongly agree”
Organizational health climate	“My organization encourages me to make suggestions about employee safety, health, and well-being”	5-point Likert scale, including “neutral”	% “agree” and “strongly agree”
Employer-provided health opportunities	“My employer has provided me with opportunities to…”		
*Physical activity*	“… be physically active”	4-point Likert scale	% “agree” and “strongly agree”
*Diet*	“… eat a healthy diet”	4-point Likert scale	% “agree” and “strongly agree”
*Tobacco consumption*	“… live tobacco free”	4-point Likert scale	% “agree” and “strongly agree”
*Stress*	“… manage my stress”	4-point Likert scale	% “agree” and “strongly agree”
*Work safety*	“… work safely”	4-point Likert scale	% “agree” and “strongly agree”
Job perceptions			
Overall satisfaction	“All in all, how satisfied would you say you are with your job?”	4-point Likert scale (“very dissatisfied” to “very satisfied”)	% “satisfied” and “very satisfied”
Recommending working at this organization	(“Overall, I would recommend working with this organization to my family and friends”)	5-point Likert scale, including “neutral”	% “agree” and “strongly agree”
Commute time	“How much time do you spend traveling to and from work each day (roundtrip)?”	5-point scale (< 15 min, 15–30 min, 30–60 min, 60–90 min, and more than 90 min)	% “ < 15 min”, “15–30 min”, and “30–60 min”

Survey items default to a 4-point Likert scale of “strongly disagree”, “disagree”, “agree”, and “strongly agree”.

### Statistical analyses

All data cleaning and statistical analyses were conducted in RStudio using R v3.6.3 (Boston, MA, USA). Percentage and mean responses to each INPUTS domain are presented in accordance with their survey manual^22^; responses are presented overall and by job site. Hypothesis testing was conducted to examine differences in item responses by job site. Pearson’s chi-squared tests were run on count data; Fisher’s exact tests were run for count data with fewer than five samples. ANOVAs were run on the continuous items (e.g., workplace safety and company health support). Due to a relatively low sample size (N = 68) and the pilot, hypothesis-generating nature of this research, two-sided *p*-values < 0.1 were considered statistically significant.

### Ethics approval

The study procedures were approved and deemed exempt by the University of Michigan Institutional Review Board (#HUM00202683).

### Consent to participate

Informed consent was obtained from all study participants. No personal identifiers were collected.

## Results

### Job demands, skill discretion, and decision authority

Of the 68 participants were recruited in this study; 36 and 15 were in the hauling and landfill divisions of the commercial multi-site company, respectively. Eight participants worked at a county recycling facility and nine worked in a local, small business. Demographics of the 68 participants are detailed open access elsewhere. Briefly, of the recruited participants, the majority were male (87%) with a a high school diploma or GED (90%), between 35 and 54 years of age (40%), and most were relatively experienced in the industry^24^. Table 2 presents the self-reported job demands, skill discretion, decision authority—widely accepted to mirror the psychosocial work environment^25^—and ergonomic risk of the participants, overall and by job site. One in 10 workers reported low psychological demands, while 3 in 4 reported high decision authority during their work shift. Similarly, 76% reported high skill discretion, although the percentages were not significantly evenly distributed by job site. Nearly all landfill workers (90%) reported high skill discretion, while just over half (56%) reported in the county site. Workers reported high ergonomic risk; 78% reported frequent, repetitive, and forceful hand movements, and 75% reported repeated lifting, pushing, pulling, or bending. Landfill workers reported the lowest ergonomic risk for both hand movement and material handling (47% and 53%, respectively).

**Table 2:** Self-reported job demands, skill discretion, and decision authority of solid waste workers overall and by job site.

Domain	Overall (N = 68) (%)	Multi-site		Single site	
		Hauling (N = 36) (%)	Landfill (N = 15) (%)	County (N = 8) (%)	Small business (N = 9) (%)
High decision authority	75	75	73	62	89
Low psychological demands	10	11	7	25	0
High skill discretion*	76	76	90	56	72
Low ergonomic risk					
Hand movement**	22	19	47	12	0
Material handling**	25	17	53	0	33

*p < 0.1; **p < 0.05; ***p < 0.01.

### Interpersonal relationships

Most participants reported high supervisor support (76%), with slightly fewer workers reporting high coworker support (64%) (Table 3). Less than half of the workers (45%) reported a caring coworker work environment. Meanwhile, more than half of workers (59%) reported some conflict at home from issues at work; conflict at work from issues at home was less prevalent (43%). Responses were not significantly different across job sites; however, county workers reported lower coworker support (43%) and more work-family conflict (75%) compared to other job sites.

**Table 3:** Self-reported interpersonal relationships of solid waste workers overall and by job site.

Domain	Overall (N = 68) (%)	Multi-site		Single site	
		Hauling (N = 36) (%)	Landfill (N = 15) (%)	County (N = 8) (%)	Small business (N = 9) (%)
Coworker support	64	68	67	44	61
Supervisor support	76	69	83	88	78
Lack of work-family conflict	41	47	33	25	44
Lack of family-work conflict	57	64	47	62	44
Coworker health climate	45	44	47	44	44

### Organizational health and safety factors

Just over 3 in 4 workers reported their management considered workplace health and safety to be important, with the non-commercial sites reporting the highest prevalence (Table 4). On the other hand, organizational health was low; only 26% of workers reported their organization encouraging them to make suggestions about employee safety, health, and well-being. On a scale from 1 to 10 (10 being extremely supportive), participants on average reported their companies to be somewhat supportive of their personal health (6.1), with 51% reporting their supervisor encouraging healthy behaviors. Overall, most workers reported their employer providing them with the opportunity to be physically active (79%) and to work safely (59%). Less than 1 in 3 workers reported their employer providing opportunities to eat a healthy diet (29%), live tobacco free (29%), or manage their stress (25%). Opportunities to be physically active were significantly different by job site; only 33% of landfill workers reported as such, while all county workers reported so.

**Table 4:** Self-reported organizational health and safety factors of solid waste workers overall and by job site.

Domain	Overall (N = 68) (%)	Multi-site		Single site	
		Hauling (N = 36) (%)	Landfill (N = 15) (%)	County (N = 8) (%)	Small business (N = 9) (%)
Management health and safety support	76	72	73	88	89
Company health support	6.1	6.4	5.8	4.1	6.3
Supervisor health support	51	56	67	25	33
Organization health climate	26	17	40	38	33
Employer-provided health opportunities					
Be physically active***	72	81	33	100	78
Eat a healthy diet	29	22	40	38	33
Live tobacco free	29	28	47	12	22
Manage my stress	25	22	27	12	44
Work safely	59	53	73	62	56

*p < 0.1; **p < 0.05; ***p < 0.01.

### Overall job perceptions

**Figure 1:**
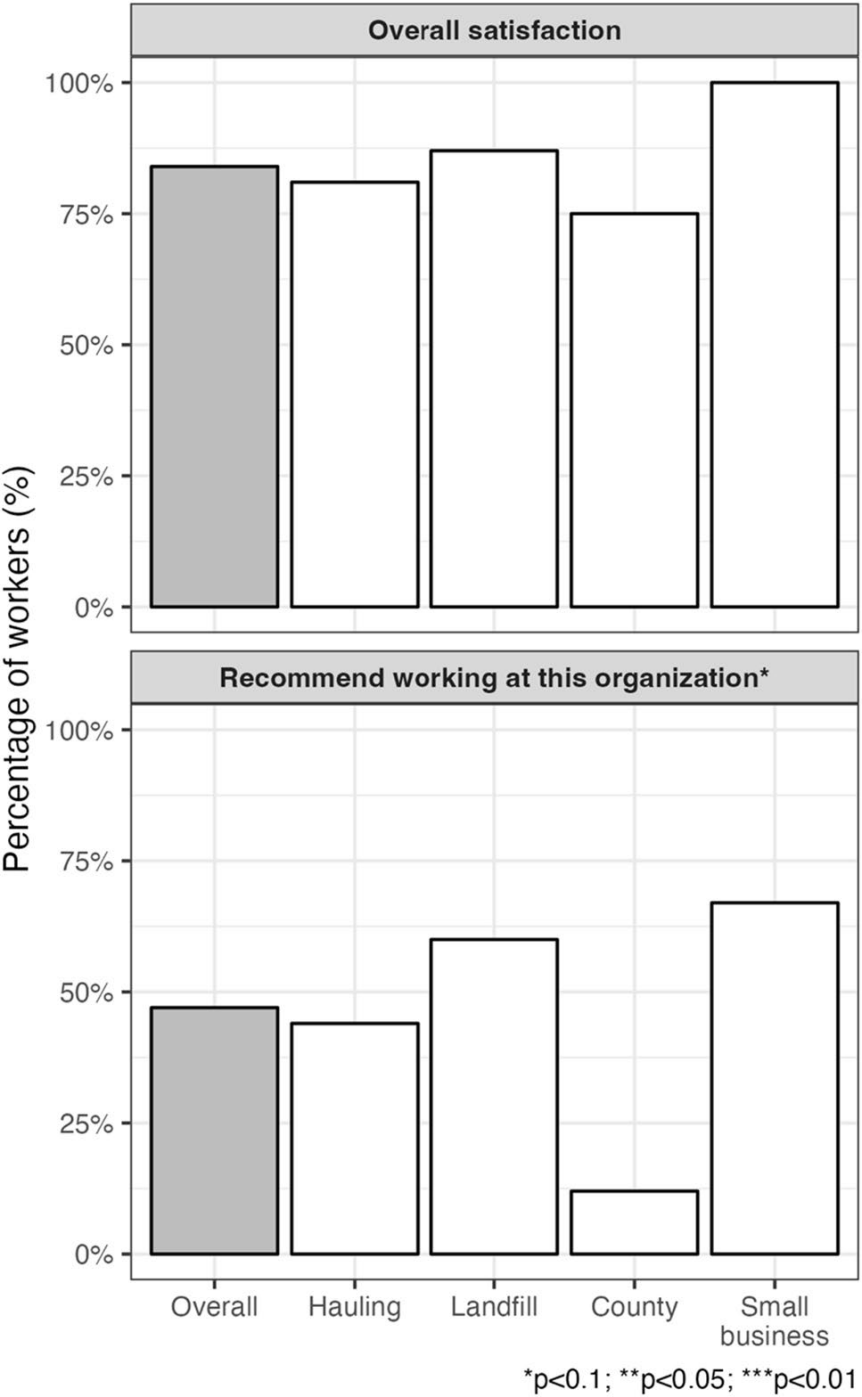
Self-reported job perceptions of solid waste workers overall and by job site.

Overall job satisfaction was high among the workers (84%) and was similar by job site, although the local, small site workers all reported job satisfaction (Fig. 1). Roundtrip, most workers spent less than 1 h commuting to and from work each day (71%). On a scale from 1 to 10 (10 being extremely safe), workers on average perceived their workplace as somewhat safe (6.5), with little difference by job site. Less than half (47%) reported that they would recommend working with this organization to their family and friends, although this was significantly different by job site. The lowest prevalence of recommendation was at the county recycling site (12%), while most of the local, small site and landfill workers would give a recommendation (67% and 60%, respectively).

## Discussion

This cross-sectional pilot study explored perceptions of psychosocial factors of work and organizational health among a sample of U.S. solid waste workers. Overall, the workers in this study reported a generally positive psychosocial work environment with low psychological demands, high skill discretion, and high decision authority. However, skill discretion was highest among landfill workers compared to their counterparts in hauling and single sites. Landfill workers generally function autonomously operating a single-cabin compactor (e.g., wheel dozer) and had the most predictable workdays while those who work in hauling do not remain in a single location all day and have less predictable day-to-day activities and interactions. While more than 80% of workers reported job satisfaction, most did not recommend their line of work to others; this may be due to the job demands that negatively impact psychosocial health. A majority of the workers reported high ergonomic risks, with repetitive motion and material handling of dynamic loads. This can make them susceptible to cumulative trauma disorders^26^ and musculoskeletal disorders (MSDs)^27^, which can lead to chronic pain if not properly addressed. The bidirectional relationship between poor psychosocial factors, such as low job satisfaction, high job demands, and perceived lack of control over the work environment and MSDs are well documented in the literature^28–31^. However, since participants in this study did not report a negative psychosocial environment, addressing MSD risks via traditional approaches to addressing ergonomic hazards (e.g., stretching, ergonomic program, automation) may be more impactful than focusing on altering the psychosocial environment.

Regarding interpersonal relationships, the majority of workers reported sufficient having co-worker and supervisor support—key buffers to workplace stress^32–34^. However, nearly two-thirds and half of participants reported work-family conflict (WFC) and family-work conflict (FWC), respectively. While no study has been conducted on WFC or FWC in U.S. waste workers, a study on perceived stress and psychosocial factors among e-waste workers in West Africa found that perceived work stress increased when there was work interference with family responsibilities or leisure time due to over-involvement, longer commute times, or exceeding regular working hours—this implication may be generalizable beyond the waste industry as well^35^. While the workers of the e-waste study were informal workers compared to formal solid waste workers of this present study so it is not a one-to-one comparison, there are similarities in the demands of the nature of the work (e.g., type of physical labor, outdoor exposures, overtime). Moreover, the lack of additional studies to cite on the psychosocial factors of work among waste workers is further evidence of the need of our study to highlight this overlooked population.

Most workers believed their management to support OSH measures but less so regarding other domains of health, such as individual diet and lifestyle factors. The concept of Total Worker Health (TWH)—a holistic approach to worker well-being that recognizes work as a social determinant of health^36^—has been slower to be embraced in workplace settings with more workers engaged in manual labor^37–39^. This may be in part due to the presence of addressing more acute physical hazards taking precedence (e.g., preventing injuries, lacerations, slips and trips), a lack of understanding of social determinants of health, differences in health behaviors and lifestyle of manual labor workers versus office workers, and/or dissimilarities in uptake of workplace health promotion/interventions^37–39^. However, it may be net beneficial for industries like solid waste management to familiarize themselves with TWH and to begin to adopt some TWH practices into their workplace as it has not only demonstrated a return-on-investment in terms of reduction of workplace injuries and illnesses, but also improved psychosocial factors of work and perceptions of organizational health that help retain workers and keep them satisfied and productive^36^.

### Limitations and strengths

This study has several limitations. First, this study was cross-sectional so the findings may be subject to temporal bias in workers responses due to factors such as seasonal changes in workload and especially since this survey was administered during the first year of the COVID-19 pandemic when the nature of work shifted drastically for much of the population. Second, the sample size of this pilot study was relatively small and may not be representative of all U.S. solid waste workers. Third, participation in this study may have been limited due to one activity (not reported here) involving saliva collection; given the timing of the study—early in the COVID-19 pandemic—people may have been hesitant to participate in research studies that involved the collection of biological specimens. Fourth, there is potential selection bias because site supervisors had to consent to the study team sampling their workers, which may bias the findings towards more positive responses to workplace psychosocial factors and organizational health. Fifth, the study population may not be generalizable due to geographic location and only one site of each type being sampled. Sixth, the survey instrument operationalized was not intended to compare individual responses but rather aggregate scores by company/job title/etc. This limits its applicability in research, particularly for occupational epidemiology studies, but for the purpose of our study—to characterize organizational health—it was appropriate. Despite these limitations, strengths of this study include being the first, to our knowledge, to assess psychosocial factors of work and perceptions of organizational health among U.S. solid waste workers—an overlooked but essential worker population.

## Conclusions

The descriptive analyses in this pilot study suggest that solid waste workers in the U.S. have both a positive psychosocial work environments and strong organizational health. However, given the limited scope of this study, future studies on U.S. waste workers are needed, especially longitudinal, to provide stronger evidence for these findings and to understand potential heterogeneity in psychosocial factors and organizational health across U.S. waste sites. Furthermore, more data needs to be collected on the health and lifestyle factors of U.S. waste workers to better characterize the extent that occupation impacts physical and mental health for workers in this essential industry.

## Data Availability

The data analyzed during the current study are available from the corresponding author on reasonable request.
